# Cutaneous Angiosarcoma of the Scalp: A Case Report of Sustained Complete Response Following Liposomal Doxorubicin and Radiation Therapy

**DOI:** 10.1080/13577140500043948

**Published:** 2005

**Authors:** Caroline L. Holloway, A. Robert Turner, George S. Dundas

**Affiliations:** 1 Division of Radiation Oncology Department of Oncology University of Alberta Edmonton Alberta Canada; 2 Division of Medical Oncology Department of Oncology University of Alberta Edmonton Alberta Canada; 3 Department of Radiation Oncology Cross Cancer Institute 11560 University Avenue Edmonton Alberta Canada

## Abstract

Cutaneous angiosarcomas of the head and neck are aggressive cancers with a mean overall survival of 30 months. We add to the literature a case report of a 65-year-old man with a large, >10 cm, unresectable, angiosarcoma of the scalp who was treated with two cycles of liposomal doxorubicin (Caelyx®) followed by electron beam radiation therapy (30 Gy in 10 fractions over 2 weeks) who has sustained a complete response with a 4-year follow-up. The dose and fractionation of the radiation therapy in this case was palliative and was not expected to give lasting local control of this lesion. It is therefore possible that either the genetic profile of the tumour conferred radiosensitivity or that the radiation therapy induced a recall phenomenon of the liposomal doxorubicin.


*Sarcoma,* March/June 2005; 9(1/2): 29–31

Taylor & Francis

Taylor & Francis Group

**CASE REPORT**

### Cutaneous angiosarcoma of the scalp: A case report of sustained complete response following liposomal doxorubicin and radiation therapy

CAROLINE L. HOLLOWAY[1], A. ROBERT TURNER[2], & GEORGE S. DUNDAS[1]

*Division of [1]Radiation Oncology, and [2]Medical Oncology, Department of Oncology, University of Alberta, Edmonton, Alberta, Canada*

**Abstract**
Cutaneous angiosarcomas of the head and neck are aggressive cancers with a mean overall survival of 30 months. We add to the literature a case report of a 65-year-old man with a large, >10 cm, unresectable, angiosarcoma of the scalp who was treated with two cycles of liposomal doxorubicin (Caelyx®) followed by electron beam radiation therapy (30 Gy in 10 fractions over 2 weeks) who has sustained a complete response with a 4-year follow-up. The dose and fractionation of the radiation therapy in this case was palliative and was not expected to give lasting local control of this lesion. It is therefore possible that either the genetic profile of the tumour conferred radiosensitivity or that the radiation therapy induced a recall phenomenon of the liposomal doxorubicin.

**Keywords:** *Cutaneous angiosarcoma, liposomal doxorubicin, radiation therapy*

### Introduction

Angiosarcoma of the head and neck is an uncommon aggressive cancer of the skin and soft tissues [1]. They occur most commonly in the sixth to seventh decade in males and may be related to previous radiation exposure or chronic lymphedema [2], but are often spontaneous. The size of the tumour has been found to be a prognostic indicator [3], with patients having lesions <10 cm surviving longer than patients with tumours >10 cm. The optimal treatment for angiosarcomas of the skin involves complete surgical resection, followed by local radiation therapy [4–6]. If the tumour is too extensive to consider surgical resection, there is no standard treatment. Palliative chemotherapy alone results in a mean survival of 7.5 months [7] and palliative radiation therapy results in a mean survival of 13.5–15 months [8].

There has been a report of regression of angiosarcomas of the skin with the use of liposomal doxorubicin [9]. We report a case of a 65-year-old man with a large unresectable angiosarcoma on the scalp who was treated with two courses of liposomal doxorubicin (Caelyx®) prior to receiving electron beam radiation therapy (3000 cGy/10 fractions) and who has had a sustained remission.

### Case report

A 65-year-old male presented with a 1-year history of a progressive bruise-like lesion with associated punctate infarcts on the forehead above the glabella. The initial biopsies showed evidence of lymphedema thought to be secondary to complications from his previously diagnosed temporal arteritis. Further biopsies showed infiltrating atypical vascular channels with evidence of hemorrhage and were positive for CD31 and CD34 immunohistochemistry stains. A diagnosis of cutaneous angiosarcoma was made and this was reviewed and confirmed by our sarcoma pathologist.

A bone scan was obtained, as were CT scans of the head/neck, thorax and abdomen. There were no distant metastases nor evidence of skull involvement.

Correspondence: Dr Caroline Holloway, Department of Radiation Oncology, Cross Cancer Institute, 11560 University Avenue, Edmonton, Alberta, Canada T6G 1Z2. Tel: +1-780-432-8754. Fax: +1-780-432-8380. E-mail: carolhol@cancerboard.ab.ca
Abstract submitted for poster presentation at Canadian Association of Radiation Oncologists (CARO), September, 2004, Halifax, Canada.

ISSN 1357–714X print/ISSN 1369–1643 online © 2005 Taylor & Francis Group Ltd
DOI: 10.1080/13577140500043948

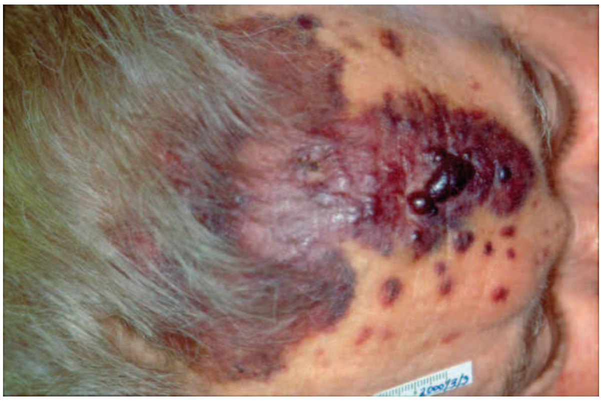

Figure 1. Cutaneous angiosarcoma of scalp: pre-treatment.

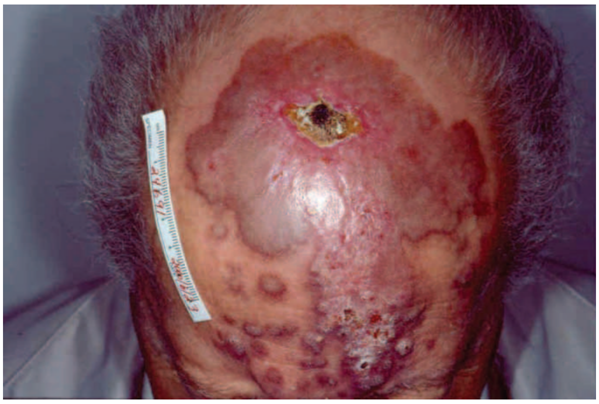

Figure 2. Progression of cutaneous angiosarcoma following two cycles of Caelyx®.

The lesion on the forehead was a 15 × 12 cm plaque with several satellite red papules (Figure 1).

His past medical history included temporal arteritis/polymyalgia rheumatica, and abdominal aortic aneurysm as well as a high-risk prostate adenocarcinoma for which he was on leuprolide injections.

*Treatment*

The lesion was considered too extensive for surgical resection. Based on a previous report it was decided to start the patient on Caelyx® 20 mg/m$^2$ (50 mg) every 4 weeks [9]. Three weeks after the first cycle the lesion was noted to be flatter. It was then decided to increase the dose to 40 mg/m$^2$ (100 mg), a dose commonly used in the metastatic breast cancer setting. Unfortunately, following this second course, he developed severe mucositis and palmar plantar erythrodysesthesia, a known, dose-related, complication of liposomal doxorubicin [10]. As well, he was noted at this time to have progression of his lesion with periorbital edema and further skin involvement down the nasal bridge (Figure 2). It was elected to proceed with radiation therapy with palliative intent. The patient was treated supine in a perspex shell for immobilisation. Bolus (0.5 cm) was applied over the shell. An irregular electron cut-out was used to define the treatment area. A single superior/anterior oblique field of 9 MeV electrons was used to deliver the prescribed dose of 3000 cGy to the 90% isodose curve in 10 fractions (3 Gy/fraction) over 2 weeks.

*Follow-up*

As early as the first week following the completion of the radiation therapy there was evidence of tumour flattening and formation of eschars. At one month follow-up it was noted that there was significant fading of the tumour area and that the lesion had flattened. Continued improvement with further fading of the pigmented areas has occurred over the ensuing months. The patient has now been followed for 4 years and shows no evidence of disease recurrence despite the palliative intention and doses of the radiation therapy (Figure 3).

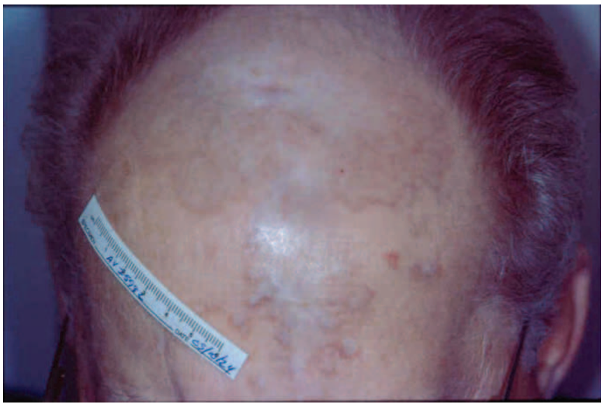

Figure 3. Sustained complete response 46 months post treatment.

### Discussion

Angiosarcomas of the skin are aggressive tumours with high rates of recurrence and metastasis. The mean overall survival with surgical resection is 30 months [1]. If surgical resection is not feasible there currently is no standard treatment. Recent case reports have shown promising results utilising liposomal doxorubicin [9] as well as combined radiation therapy with liposomal doxorubicin [11,12].

Pegylated liposomal doxorubicin (Caelyx®) has been shown to be effective in the treatment of Kaposi's sarcoma [13] and is being investigated for use in metastatic breast cancer as well as T-cell leukemia and gynecological malignancies [10,14]. Pegylated liposomal doxorubicin has a long serum half-life and is able to accumulate in tumour tissues. As liposomal doxorubicin has less 'free' doxorubicin in the serum compared with doxorubicin, there is less cardiotoxicity and myelotoxicity seen [10,15].

Doxorubicin has been shown to be a radiosensitizer, *in vitro*, at higher serum concentrations [16]. However, *in vivo* the response has been more variable and may in fact increase tumour hypoxia and therefore decrease the response to radiation if given immediately prior to radiation therapy [17]. In this case a higher dose of Caelyx® (100 mg) was given 1 month prior to the patient receiving radiation therapy. Although, there was evidence of tumour progression following the Caelyx®, it may have acted by increasing the tumour radiosensitivity.

This case report demonstrates a sustained complete response of cutaneous angiosarcoma after two cycles of Caelyx® followed by high dose per fraction (30 Gy /10 fractions) radiation therapy. Previous case reports had illustrated response to liposomal doxorubicin following failed radiation therapy 40–60 Gy (2 Gy/fraction) [11,12]. The dose and fractionation of radiation therapy in this case, as it was palliative in intent, would not be presumed to have lasting local control of the lesion. It is possible that the genetic profile of the tumour conferred radiosensitivity or if the anthracycline was incorporated into the DNA of the tumour, we may have seen a 'chemotherapy' recall with the subsequent radiation therapy.

### References

1. Fedok FG, Levin RJ, Maloney ME, Tipirneni K. Angiosarcoma: current review. *Am J Otolaryngol* 1999;20:223–231.
2. Cooper PH. Angiosarcomas of the skin. *Semin Diagn Pathol* 1987;4:2–17.
3. Lydiatt WM, Shaha AR, Shah JP. Angiosarcoma of the head and neck. Am J Surg 1994;168:451–454.
4. Brand C, Yawalkar N, von Briel C, Hunziker T. Combined surgical and X-ray treatment for angiosarcoma of the scalp: Report of a case with a favourable outcome. *Br J Dermatol* 1996;134:763–765.
5. Bullen R, Larson P, Landeck A, *et al.* Angiosarcoma of the head and neck managed by a combination of multiple biopsies to determine tumor margin and radiation therapy. Report of three cases and review of the literature. *Dermatol Surg* 1998;24:1105–1110.
6. Mark R, Tran L, Sercarz J, Fu Y, Calcaterra T, Juillard G. Angiosarcoma of the head and neck. The UCLA experience 1955 through 1990. *Arch Otolaryngol Head Neck Surg* 1993;119:973–978.
7. Maddox J, Evans H. Angiosarcoma of the skin and soft tissue. A study of forty-four cases. *Cancer* 1981;48: 1907–1921.
8. Holden CA, Spittle MF, Jones EW. Angiosarcoma of the face and scalp, prognosis and treatment. *Cancer* 1987;59: 1046–1057.
9. Ockenfels H, Brockmeyer N, Hengge U, Goos M. Cutaneous angiosarcoma: A novel therapy with liposomal doxorubicin? *J Eur Acad Dermatol Venereol* 1996;6:71.
10. Cattel L, Ceruti M, Dosio F. From conventional to stealth liposomes: A new frontier in cancer chemotherapy. *Tumori* 2003;89:237–249.
11. Wollina U, Fuller J, Graefe T, Kaatz M, Lopatta E. Angiosarcoma of the scalp: treatment with liposomal doxorubicin and radiotherapy. *J Cancer Res Clin Oncol* 2001;127:396–399.
12. Eiling S, Lischner S, Busch JO, Rothaupt D, Christophers E, Hauschild A. Complete remission of a radio-resistant cutaneous angiosarcoma of the scalp by systemic treatment with liposomal doxorubicin. *Br J Dermatol* 2002;147:150–153.
13. Dezube BJ. Management of AIDS-related Kaposi's sarcoma: advances in target discovery and treatment. *Expert Rev Anticancer Ther* 2002;2:193–200.
14. Wollina U, Dummer R, Brockmeyer NH, *et al.* Multicenter study of pegylated liposomal doxorubicin in patients with cutaneous T-cell lymphoma. *Cancer* 2003;98:993–1001.
15. Gabizon A, Catane R, Uziely B. Prolonged circulation time and enhanced accumulation in malignant exudates of doxorubicin encapsulated in polyethylene-glycol coated liposomes. *Cancer Res* 1994;54:987–992.
16. Durand RE. Adriamycin: a possible indirect radiosensitizer of hypoxic tumor cells. *Radiology* 1976;119:217–222.
17. Durand RE, LePard NE. Tumour blood flow influences combined radiation and doxorubicin treatments. *Radiother Oncol* 1997;42:171–179.

